# Supplementary material for: Comparison of two popular transducers to measure sit-to-stand power in older adults
Source: PLoS One. 2024 Aug 12;19(8):e0308808. doi: 10.1371/journal.pone.0308808 (PMC11318872; doi:10.1371/journal.pone.0308808)
Supplement: Attachment — Submitted filename: Response to Reviewers.docx [file pone.0308808.s001.docx]

We thank all the reviewers for their generous comments and suggestions, which have considerably improved the quality of the paper. Our revisions in the paper are highlighted in red.

**Reviewer #1:**
1.This paper compares for the first time two common methods to assess muscle power in older adults, the Tendo Unit (TU) and the Gym Aware (GA). As a second aim, it confirms the correlation between the STS peak power and physical performance measures AND LP power. The findings are positive, with a high degree of agreement and similar correlations with physical function.
The study is based on a solid statistical method that includes a sample size estimate prior to data collection. Such occurrence makes it hard to point out that my only comment is that I would have loved to see the results of such a precise and jolly design and method on a larger sample with age stratification and frailty stratification. Other than that, I don't have major nor minor comments to submit.

We thank you profusely for the very positive comment and pointing out the pre-registration of the sample size and analysis plan.

We wholeheartedly agree with your comment about having a larger sample size with a spectrum of participants from different age groups and functional status. We have added this comment in our limitations section: “...the majority of our participants could be classified as high-functioning with an average age of 70 years, meaning these results may not be extrapolatable to low-functioning older adults or those outside the average age group.”

2.(watch out for the typing error in the title)

We apologize for the error. We have now corrected the title. Thank you once again.

**Reviewer #2:**
1.The study by Balachandran et al. compared two popular assessment tools for skeletal muscle power in geriatric patients. The study is carefully done, and the results are sound and well-presented. All conclusions are supported by the data. There are certainly some limitations to this study that are adequately detailed in the discussion section. However, the manuscript needs minor improvements:

Thank you so much for the very positive comments. We do appreciate it.

2.Page 18, line 360: The majority of our participants were high-functioning.
The authors are kindly asked to provide this information in material and methods under inclusion criteria. What does high functioning mean precisely?

The functional or performance status was based on the SPPB test, which is a composite measure of three performance tests: walk speed, balance, and chair stand. SPBB categorizes functional or physical performance status based on the total score obtained with 10-12 points considered as “high functioning”. The SPBB test is widely used in large multicenter RCT’s to screen for functional/ physical performance status in older adults.

We did not have an inclusion criterion based on the functional status and our intention was just to report the functional/physical performance status of our participants for the readers. We have added the following line to add clarity: “Based on the average SPPB score of 11.3, the majority of our participants could be classified as high-functioning [42]...”

We have now included a reference for these cut-offs in the paper: Guralnik JM, Ferrucci L, Simonsick EM, Salive ME, Wallace RB. Lower-extremity function in persons over the age of 70 years as a predictor of subsequent disability. N Engl J Med. 1995;332: 556-562.

Page 18, lines 356-357: we chose to use both transducers simultaneously, rather than on separate occasions. Indeed, this is an important point. Pneumatic leg press, Gym Aware and Tendo Unit, SPPB, and Timed up and go (TUG) were separately explained on pages 6-9. The authors should provide detailed information of the sequence of assessments. For instance, were all assessments performed at the same occasions? Were all assessments arranged in the same order for all participants?

Thank you for the excellent suggestion. All assessments were performed on the same occasion on the same day. We have added this line right above the paragraphs about tests to clarify the sequence of testing: “All the following tests for all the participants were conducted in the order given below:”.

We once again thank the reviewers for their valuable suggestions and extremely positive comments. :)
